# Effects of participation in interdisciplinary rounds in the intensive care unit on family satisfaction: A cross-sectional study

**DOI:** 10.5935/2965-2774.20230274-en

**Published:** 2023

**Authors:** Daniel Schneider, Regis Goulart Rosa, Rosa da Rosa Minho dos Santos, Débora Vaccaro Fogazzi, Gabriela Soares Rech, Daiana Barbosa da Silva, Mellina da Silva Terres

**Affiliations:** 1 Universidade Federal de Ciências da Saúde de Porto Alegre - Porto Alegre (RS), Brazil; 2 Hospital Moinhos de Vento - Porto Alegre (RS), Brazil

**Keywords:** Critical care, Family, Personal satisfaction, Patient satisfaction, Survey and questionnaires

## Abstract

**Objective:**

To investigate whether family participation in intensive care unit interdisciplinary bedside rounds affects family satisfaction.

**Methods:**

A cross-sectional study was conducted at a 56-bed, adult, mixed intensive care unit of a tertiary hospital in Southern Brazil. From May to June 2019, family members of patients who stayed in the intensive care unit for at least 48 hours were invited to participate in the study at the time of patient discharge. The main exposure variable was participation in intensive care unit bedside rounds during the intensive care unit stay. Family satisfaction was assessed by using the Brazilian version of the Family Satisfaction in the Intensive Care Unit questionnaire.

**Results:**

Of the 234 screened individuals, 118 were included. Eleven participants withdrew consent. A total of 107 individuals were assessed; 58 (54%) reported being present during bedside rounds, and 49 (46%) reported never being present. General satisfaction and satisfaction with the decision-making process were higher among families who were present during rounds than among families who were not (p = 0.01 and p = 0.007, respectively).

**Conclusion:**

The presence during interdisciplinary rounds was associated with improved general satisfaction and satisfaction with the decision-making aspect. This outcome indicates that efforts must be directed to conduct studies with more robust methodologies to confirm this association.

## INTRODUCTION

Meeting the needs of patients and family members and integrating their values into the care provided is considered a core aspect of quality in critical care.^(1-3)^ Patients admitted to the intensive care unit (ICU) are frequently limited in their ability to participate in decision-making. In this context, family members are generally required to act as surrogate decision-makers,^(4-6)^ and their satisfaction may be considered a proxy of the evaluation and expectations of the patient. In the last two decades, several studies have focused on family satisfaction in the ICU.^(7)^ Among these studies, communication between family members and the ICU staff has been considered a predominant aspect.^(1,2,8-11)^

The inclusion of family members in ICU interdisciplinary rounds has been proposed and shown to improve communication and satisfaction.^(6,12-16)^ This practice is defined as family members being present in the patient’s room during rounds and represents an opportunity for promoting shared decision-making.^(6,17)^ In a scenario of uncertainty regarding patient survival, families desire easy access to comprehensive information^(18)^ and more frequent communication with the ICU team,^(9)^ which may be supplied by including them in interdisciplinary rounds. In addition, there is evidence that contradictory information and failure to understand clinical information are associated with decreased family satisfaction.^(11,19,20)^ Despite being recommended by the Society for Critical Care Medicine (SCCM),^(3,21)^ health care providers have concerns about including family members in rounds since it may prolong the rounding time, limit the details of the discussion and increase family stress.^(12,22-24)^ Although many studies address the presence of family members during interdisciplinary rounds in pediatric ICUs, data on this practice in adult ICUs are still scarce.^(14,25)^

A cross-sectional study was performed with the aim of evaluating whether the presence of family members during interdisciplinary ICU bedside rounds is associated with better satisfaction.

## METHODS

### Study design

From May to June 2019, a cross-sectional study was conducted in a 56-bed, adult, mixed ICU with flexible visiting hours in a tertiary hospital, during which family members of critically ill patients were interviewed at patient discharge.

Although family presence during interdisciplinary bedside rounds was not a policy of the institution following standardized procedures adapted to the lay public, this practice was largely encouraged since families were allowed to stay with patients for 12 hours per day. Additionally, at the moment of patient admission to the ICU, family members were informed about the visiting policy, which included the possibility of being present during interdisciplinary bedside rounds when they were invited.

### Patient population

All patients with planned discharge from the ICU were screened. Patients who stayed in the ICU for less than 48 hours were excluded. Family members over 18 years old who spoke Portuguese and had no evident cognitive limitations in responding to the interview were invited to participate. Family members were defined as all individuals who visited the patient in the ICU, regardless of their relationship to the patient. Only one respondent was included per patient. Since the study procedures included the interview performed at the time of discharge from the ICU, family members of patients who passed were not included to respect their bereavement process.

At the time of inclusion, the objectives of the study were described, individuals were informed that their participation was voluntary and anonymous, and informed written consent was obtained. The Institutional Review Board (IRB) of *Hospital Moinhos de Vento* granted ethical review and approval for this study.

### Survey development and data collection

A standardized demographic questionnaire was obtained with the following data: age, sex, time of study, monthly income and relation to the patient. Additionally, family members were asked whether they were surrogate decision-makers.

Aiming to know about presence during the ICU bedside rounds, we also included a “yes or no” question in the questionnaire. In addition, the frequency of participation was assessed through a 5-point Likert scale ranging from “never” to “always”.

The validated Family Satisfaction in the Intensive Care Unit (FS-ICU) survey was used in this study to measure general satisfaction and its two subscales: the satisfaction with care and satisfaction with decision-making subscales.^(26)^ The administration of the FS-ICU tool is based on the respondent’s rating about their experience in the ICU on a scale from 1 - 5. The average of the first 13 questions corresponds to the subscale of satisfaction with care, while the last 10 questions are related to the satisfaction with decision-making subscale. Higher values indicate increased satisfaction, and the average of all questions provides the general satisfaction score of families with the ICU.

Cronbach’s α coefficient for the two subscales was .90 and .84, referring to satisfaction with care and satisfaction with decision-making, respectively. These numbers indicate good internal consistency and show a high correlation between the items in each dimension of satisfaction.

Eligible family members were invited to participate in an interview performed by a trained research assistant. After informed consent was obtained, the data collection form and a verbal explanation about how to complete it was provided to the participants. However, the research assistant responsible for inclusion remained available to support the respondents in case any difficulties occurred.

### Statistical analysis

For this observational study, the sample size was determined by the available resources. Hence, all eligible family members were enrolled during the study period. Since we aimed to assess the association between family presence during the rounds and satisfaction, we focused the analyses on the comparison of the FS-ICU score between the groups (family members who participated in the bedside rounds *versus* those who did not participate), in relation to prior research.^(9,27,28)^

Baseline characteristics are expressed by using medians and interquartile ranges for continuous variables, while categorical variables are expressed by their relative and absolute frequencies. Chi-squared tests were used to compare categorical variables between the study groups. Sample distribution was tested by using the Kolmogorov‒Smirnov test.^(29)^ To compare FS-ICU score medians, the Wilcoxon-Mann‒Whitney test was used for two samples. The median differences are presented by using a calculated 95% confidence interval, as recommended for observational studies.^(30)^ Data analysis was conducted by using R software, version 4.0.2. A two-sided 5% significance level was used for all statistical inferences.

## RESULTS

### Demographics

**Figure 1:**
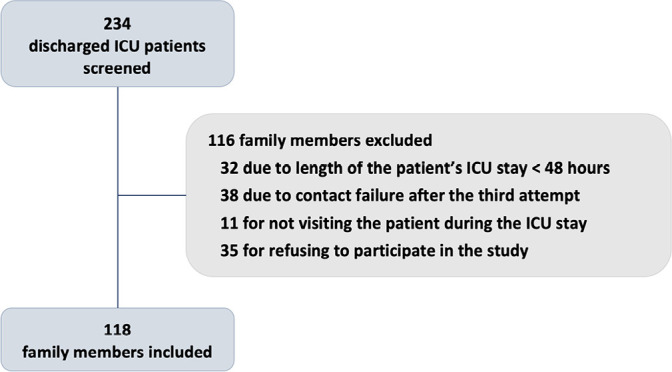
Family members inclusion.

The inclusion of participants is depicted in the flow diagram in figure 1. A total of 234 family members of patients with planned ICU discharge were screened. After the exclusion criteria were verified, 118 individuals were included. Of these individuals, 11 withdrew consent after their inclusion in the study. Thus, data from 107 family members were analyzed. The characteristics of the participating family members are presented in table 1. Among the characteristics presented in table 1, the only aspect showing a relevant difference was the sex proportion between the 58 (54.2%) family members who participated in the interdisciplinary bedside rounds and the 49 (45.8%) who did not participate.

**Table 1:** Baseline characteristics of the study participants

Characteristics of family members	All family members (n = 107)	Family members who were present during rounds (n = 58)	Family members who were not present during rounds (n = 49)
Age (years)	55.05 ± 15.99	53.96 ± 14.82	57.73 ± 15.17
Age group (years)			
< 30	6 (5.6)	4 (6,9)	2 (4.1)
30 - 49	24 (22.4)	15 (25.9)	10 (20.4)
50 - 69	43 (40.2)	24 (41.4)	19 (38.8)
70+	18 (16.9)	8 (13,8)	10 (20.4)
Not informed	16 (14.9)	7 (12.0)	8 (16.3)
Sex			
Male	23 (21.5)	9 (15.5)	14 (28.6)
Female	84 (78.5)	49 (84.5)	35 (71.4)
Education (years)	16.38 ± 6.58)	16.70 ± 7.21	16.01 ± 5.82
Monthly income (BRL)	10,000 (5,625 - 19,875)	10,500 (8,000 - 15,000)	8,000 (5,000 - 20,000)
Relationship			
Partner	47 (43.9)	29 (50.0)	18 (36.7)
Son/Daughter	35 (31.8)	17 (29.3)	18 (36.7)
Parent	11 (10.3)	3 (5.2)	8 (16.3)
Sibling	2 (1.9)	1 (1.7)	1 (2.0)
Grandchild	2 (1.9)	1 (1.7)	1 (2.0)
Other	10 (9.3)	7 (12.1)	3 (6.1)
Lives with the patient	64 (59.8)	38 (65.5)	26 (53.1)
Surrogate decision-maker	85 (79.4)	44 (75.9)	41 (83.7)
Length of ICU stay (days)	4 (3 - 5)	4 (3 - 5)	4.5 (3 - 6)

ICU - intensive care unit. The results are expressed as the mean ± standard deviation, n (%) or median (interquartile range).

ICU - intensive care unit.

### Measures of satisfaction

The median differences in the FS-ICU scores among the groups are shown in table 2. Family members who were present during the interdisciplinary bedside rounds had a significantly better general satisfaction score in comparison with family members who were not present (90.75 *versus* 82.90; median difference 7.85; 95% confidence interval [95%CI] -8.3 to -0.00005). Family presence during the rounds was also associated with increased satisfaction with decision-making (86.25 *versus* 75.00; MD, median difference 11.25; 95%CI -15.0 to -2.5). Satisfaction with care scores were not significantly different among respondents who were present during interdisciplinary rounds compared with respondents who were not. The distribution of the frequency of participation in the interdisciplinary bedside rounds, divided into “Never” (49 participants; 45.79%), “Barely” (17 participants; 15.89%), “Sometimes” (17 participants; 15.89%), “Several times” (7 participants; 6.54%), and “Always” (17 participants; 15.89%), was too heterogeneous to infer an association with the results of the satisfaction measures.

**Table 2:** Family presence during intensive care unit rounds - effects on satisfaction

Satisfaction scores	All family members (n = 107)	Family members who were present during rounds (n = 58)	Family members who were not present during rounds (n = 49)	Adjusted difference (95%CI)	p value
General satisfaction	87.5 (77.10 - 95.80)	90.75 (81.60 - 95.80)	82.90 (71.90 - 94.55)	-7.85 (-8.3 - -0.00005)	0.01^*^
Satisfaction with care	91.7 (79.20 - 98.20)	93.90 (86.05 - 98.20)	88.50 (75.0 - 98.20)	-5.4 (-11.3 - 0.99)	0.09
Satisfaction with decision-making	85.0 (68.80 - 95.0)	86.25 (77.50 - 97.50)	75.0 (64.45 - 90.0)	-11.25 (-15.0 - -2,5)	0.007^*^

95%CI - 95% confidence interval.

* Statistically significant difference considering a two-sided 5% significance level for all statistical inferences (Mann-Whitney Wilcox). The results are expressed as n (%) and median (interquartile range).

## DISCUSSION

In this cross-sectional study, the presence of family members during ICU interdisciplinary rounds, albeit not a stated policy of the institution, was associated with higher FS-ICU scores for general satisfaction and decision-making.

Although publications regarding adult ICU experiences with family presence during interdisciplinary rounds are scarce, this finding is in accordance with the available studies for both pediatric and adult care, which have shown better outcomes of satisfaction associated with the inclusion of these individuals in rounds.^(12,13,31-34)^ Notably, the literature shows that in situations of uncertainty regarding patient survival, the lack of cohesive, honest and timely information constitutes one of the main stressors for relatives of patients who are admitted to the ICU and leads to the worst satisfaction outcomes.^(1,12)^ The presence of family members during ICU interdisciplinary rounds, in which they are included in the discussion of procedures and treatment options, provides them with realistic information and a global perception of the patient’s health condition. Having such information may optimize their ability to participate in the decision-making process.^(6,25)^ Therefore, there is plausibility that the increase in general satisfaction for family members who were present during rounds was guided by the significant improvement in satisfaction with decision-making, which is probably associated with better communication with the ICU team.^(9,16)^

Conversely, satisfaction with care was not different among respondents who were present during interdisciplinary rounds and those who were not. A reason for this result is probably related to the fact that family members may collect information about care in ways other than participating in rounds, for example, during routine moments reserved to explain the patient’s clinical status or in conference rooms with physicians.^(33,35)^ Another reason for the lack of a statistically significant difference may be the fact that a calculation of the sample size was not performed, thus incurring a type II error.^(36)^ Evidence shows that the workflow in the ICU environment, which is frequently centered around technical aspects, contributes to a good perception of family members about the quality of care.^(4)^ Thus, we hypothesized that important information for the elaboration of satisfaction with care was already accessible to family members who did not participate in interdisciplinary rounds from other sources. This fact explains the similar outcomes in the participant group.

From the perspective of promoting patientand family-centered care,^(17,25)^ our findings are important because they corroborate the delivery of individualized care, respecting the beliefs and preferences of individuals.^(1,2)^ Nevertheless, the association between family presence during rounds and higher satisfaction scores is congruent with the recent scenario of studies that highlight this practice as a possible measure to promote better communication with doctors and nurses.^(9)^

This study has limitations. First, the researchers did not control family presence during the ICU interdisciplinary rounds, which was totally dependent on the ICU staff’s invitation and the family’s voluntary acceptance. Thus, the study may have incurred selection bias, as family members willing to participate in rounds were likely to have different relationships with the ICU staff and a different understanding regarding the scenario than those who chose not to participate. Data about this variable were only obtained by interviewing family members, reflecting their individual sense of participation. Thus, the number of rounds each respondent was present for could not be verified with precision. Second, our analyses did not consider variables regarding patient clinical conditions, which can influence outcomes such as family satisfaction.^(5)^ Third, this was a single-center study, which reduces the external validity of the results when compared to different scenarios. Finally, reverse causation is possible since the main exposure variable and the outcomes were assessed at the same time. Therefore, future research should focus on multicenter studies aiming to assess the influence of different ICU settings. Additionally, controlled randomized clinical trials focusing on the inclusion of family members in ICU rounds as an intervention should be conducted to provide more consistent information about the influence of communication with different ICU team members and its effectiveness.

## CONCLUSION

Family presence during interdisciplinary bedside rounds is associated with better family satisfaction outcomes. This finding is consistent and reinforces the importance of intensive care unit policy-makers to put efforts into more robust interventional trials targeting the development of safe and effective ways to include families in structured interdisciplinary rounds as an alternative way to improve satisfaction and provide appropriate support for these individuals.
